# Enzyme-linked immunosorbent assay using recombinant envelope protein 2 antigen for diagnosis of Chikungunya virus

**DOI:** 10.1186/s12985-018-1028-1

**Published:** 2018-07-24

**Authors:** Marcílio Jorge Fumagalli, William Marciel de Souza, Danillo Lucas Alves Espósito, Angélica Silva, Marilia Farignoli Romeiro, Edson Zangiacomi Martinez, Benedito Antônio Lopes da Fonseca, Luiz Tadeu Moraes Figueiredo

**Affiliations:** 1 Virology Research Center, Ribeirão Preto Medical School of University of São Paulo, Ribeirão Preto, São Paulo, Brazil; 2 Social Medicine, Ribeirão Preto Medical School of University of São Paulo, Ribeirao Preto, São Paulo, Brazil

**Keywords:** Chikungunya virus, Antibodies detection, Envelope protein 2, Serology

## Abstract

**Background:**

Chikungunya (CHIKV) virus is an important mosquito-borne virus causing outbreaks of acute febrile illness with arthropathy. The detection of specific antibodies against CHIKV is used for diagnosis after the acute viremic phase of the disease. However, a major challenge for serologic diagnosis of CHIKV and other alphaviruses is the cross-reactivity of antibodies to common antigens among these viruses. In the present study, we have developed an enzyme-linked immunosorbend assay using a recombinant envelope protein 2 of CHIKV produced in *Escherichia coli* system, as a capture antigen.

**Results:**

High titers (1600 to 12,800) of anti-CHIKV antibodies were detected in human sera analyzed by the CHIKV assay, suggesting it may detect low levels of the antibodies presence. On the other side, cross-reactivity was not observed in mouse hyperimmune sera to Mayaro virus and other alphaviruses analyzed by the CHIKV immunosorbend assay, suggesting it is a CHIKV-specific test. Fifty-nine human serum samples of CHIKV infection suspected cases were tested for immunoglobulin G (IgG) and M (IgM) antibodies detection using the CHIKV immunosorbend assay. A total of 44% (26/59) of samples were positive for IgG to CHIKV, determining 89.66% sensitivity and 100% specificity when the assay is compared to a CHIKV-specific neutralization assay. In addition, 40.6% (24/59) of samples were positive for IgM, determining 92.48% sensitivity and 79.04% specificity by a Bayesian method in the absence of a gold standard. Moreover, CHIKV immunosorbend assay showed similar sensibilities to a commercial immunochromatography assay (Lumiquick, USA) for CHIKV IgG and IgM detection.

**Conclusion:**

In short, we have developed a rapid, simple, specific and sensitive CHIKV immunosorbend assay for IgG and IgM detection and our results showed potential applicability on the diagnosis of infections by this virus.

**Electronic supplementary material:**

The online version of this article (10.1186/s12985-018-1028-1) contains supplementary material, which is available to authorized users.

## Background

Chikungunya virus (CHIKV) is included in the Semliki Forest group of the *Alphavirus* genus (*Togaviridae* family). CHIKV genome consists of a linear, positive-sense, single-stranded RNA of ~ 11.8 kb in length including two open reading frames (ORFs) that encode four non-structural proteins (nsP1–4) and five structural proteins (C, E3, E2, 6 K and E1) [1]. CHIKV has been classified into three distinct lineages named as West African, Asian and East/Central/South African (ECSA) [2].

CHIKV is a mosquito-borne virus that causes human disease mainly characterized by acute onset fever and prominent arthropathy. Humans are infected by the bite of *Aedes aegypti* and *Aedes albopictus* [3]. CHIKV infection can cause persistent arthropathy for weeks to years, leading to incapacitation of patients and substantial economic loss [4].

CHIKV was first isolated from an acute febrile human case in 1953 during a Dengue epidemic in Liteho city, Tanzania [5]. CHIKV outbreaks were initially restricted to the African continent, and had several decades of relative inactivity, re-emerging in 2005 with significant outbreaks in Africa, Asia, Europe, and in islands of Indian and Pacific Oceans [6, 7]. In late 2013, CHIKV was reported in the Americas producing outbreaks in Caribbean islands [8]. Since then, local transmission has been described in many countries throughout the Americas [9]. In 2014, CHIKV was introduced twice in Brazil, one by the Asian strain in the North region and another by the ECSA strain in the Northeast region [10]. Only during 2016 and 2017, more than 460,000 suspected cases of CHIKV were reported in Brazil, leading to at least 383 deaths [11].

CHIKV is an important public health problem in the Americas requiring early and accurate diagnosis of infections for a proper health care of patients and adoption of adequate preventive procedures. Currently, CHIKV is diagnosed by using a Real time quantitative polymerase chain reaction (RT-qPCR). However, this assay allows detection only in early viremic phase, which typically lasts up to 6 days after disease onset [12]. The confirmation of CHIKV infection after viremic phase requires serological tests. Commercial and in-house serological methods have been reported, including those based on CHIKV native antigens [13] and those using recombinant antigenic proteins [14, 15]. Most of these assays detect CHIKV specific and other alphavirus cross-reactive antibodies, evidencing low specificity. Additionally, the performance of commercially available rapid tests and the majority of Mac-ELISAs for antibody detection have shown low sensitivity [16, 17].

Herein, we show an ELISA using recombinant envelope protein 2 (rE2) of CHIKV as antigen. The rE2 of CHIKV expressed in *Escherichia coli* system was used in ELISA to detect IgG and IgM antibodies to CHIKV. To determine their sensitivities and specificities, results obtained with these assays were compared to those obtained with other routinely used serologic tests for diagnosis of infections by CHIKV.

## Results

### Standardization of rE2-CHIKV ELISA

**Fig. 1:**
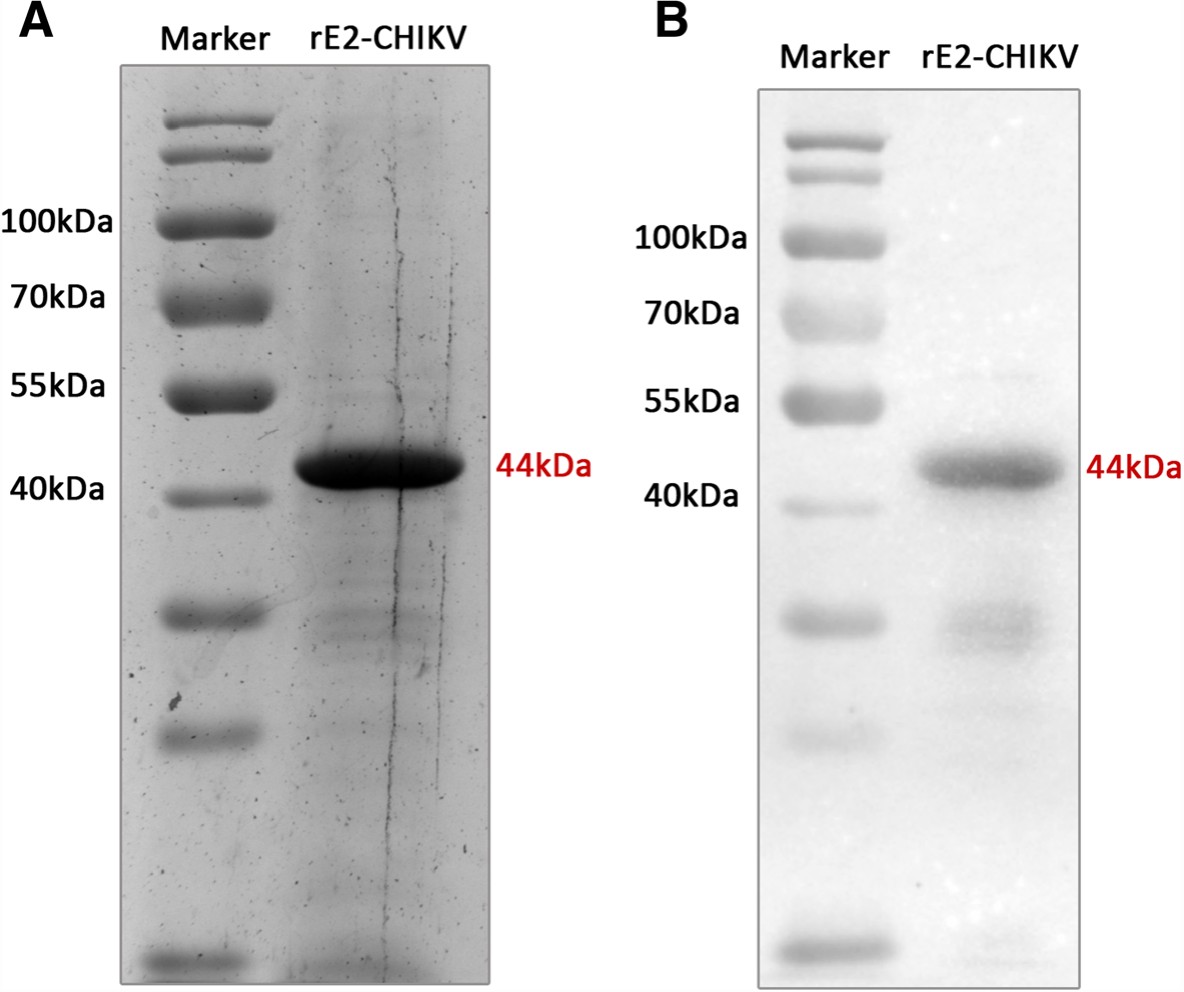
Recombinant rE2-CHIKV antigen. **a**) SDS-PAGE showing the ~ 44 kDa band of rE2-CHIKV. **b**) Western blot using mouse monoclonal anti-his antibody conjugated to horseradish peroxidase (Sigma, USA)

**Fig. 2:**
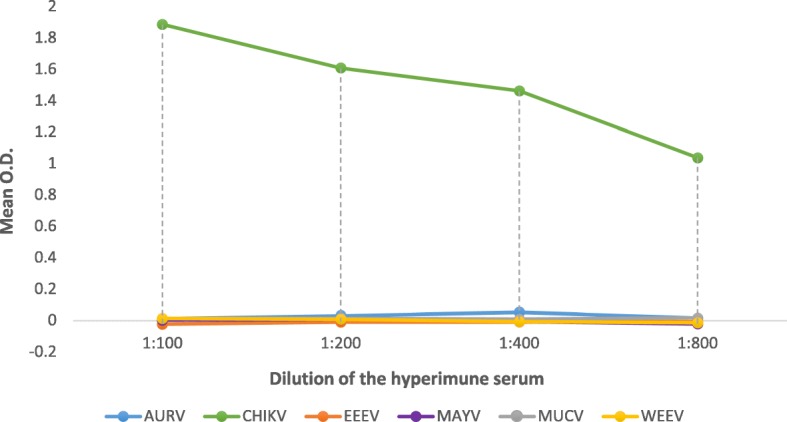
Cross-reactivity of alphavirus antibodies in the rE2-CHIKV ELISA. Cross-reaction of mouse alphavirus hyperimmune serum of AURV, EEEV, MAYV, MUCV, and WEEV (also including CHIKV as positive control) in the rE2-CHIKV ELISA

A suitable amount of 44 kDa rE2 of CHIKV was produced in *E. coli* cells, and its purity was confirmed by sodium dodecyl sulfate polyacrylamide gel electrophoresis (SDS-PAGE) and Western blot (Fig. 1a and b). The optimal concentration of recombinant rE2-CHIKV for coating ELISA plate wells was determined based on a clear detection of anti-CHIKV antibodies using mouse hyperimmune serum, which was 10 times higher than the cut off. Higher concentrations of rE2 were not chosen because they did not increase the assay sensitivity. Thus, 1 μg/ml of rE2-CHIKV per well after 18 h of coating incubation were chosen as suitable for the assay. Importantly, cross-reactivity of other mouse hyperimmune sera was not observed for alphaviruses, such as AURV, EEEV, MAYV, MUCV, and WEEV in the rE2-CHIKV ELISA (Fig. 2).

### Evaluation of rE2-CHIKV ELISA

Results obtained by rE2-CHIKV in IgG and IgM ELISAs analyzing 59 serum samples of CHIKV suspected cases were compared to those obtained using enzyme immunoassay with infected cultured cells (EIA-ICC), commercial immunochromatography assay and plaque reduction neutralization test of 50% of plaques (PRNT_50_) (Table 1 and Additional file 1: Table S1). The IgG rE2-CHIKV ELISA showed a sensitivity of 89.66% and a specificity of 100% when compared to PRNT_50_ results, considering 80 as a titer cutoff. Importantly, all 26 positive results in rE2-CHIKV ELISA were also positive by PRNT_50_, and three false negative results were observed. The comparison of IgG CHIKV PRNT_50_ results with those obtained with CHIKV EIA-ICC and commercial CHIKV immunochromatography assay showed 82.76 and 89.66% sensitivity and 96.67 and 100% specificity, respectively (Table 2). Additionally, IgG positive serum samples in the rE2-CHIKV ELISA showed 73% of high, 8% of medium and 19% of low avidity (Additional file 1: Table S1). The Bayesian analysis estimated a sensitivity of 92.48% and specificity of 79.04% for the IgM rE2-CHIKV ELISA. Sensitivities and specificities for IgM detection of the EIA-ICC were 74.01 and 55.59% and for the Commercial assay were 91.31 and 94.21%, respectively (Table 3). In addition, IgM positive samples showed 25% of high, 29% of medium and 46% of low avidity (Additional file 2: Table S2).

**Table 1:** Number of positive and negative samples detected by each of the assays

	IgG detection			PRNT_50_	IgM detection		
	rE2-ELISA	EIA-ICC	Commercial Assay		rE2-ELISA	EIA-ICC	Commercial Assay
Positive	26	25	26	29	34	37	11
Negative	33	34	33	30	25	22	48

**Table 2:** Analysis the assays regarding IgG detection of human serum samples compared to PRNT_50_ for CHIKV

Assay	Results for IgG detection			
	Positive samples	Negative samples	Sensitivity (%)	Specificity (%)
PRNT_50_	29	30	–	–
rE2-CHIKV	26	33	89.66%	100.00%
EIA-ICC	25	34	82.76%	96.67%
Commercial Assay	26	33	89.66%	100.00%

**Table 3:** Prior distribution estimates and Bayesian posterior distribution estimates for IgM sensitivities and specificities of the assays

	Prior distribution estimate				Posterior distribution estimate	
	Mode	UL^a^	LL^b^	Beta	Median	95% PI
Sensitivity						
rE2-CHIKV ELISA	0.9285		0.90	100, 8.623	0.9248	0.8671–0.9638
EIA-ICC	0.4260	0.75		1.235, 1.317	0.7401	0.4345–0.9478
Commercial Assay	0.9231		0.90	100, 9.247	0.9131	0.9131–0.9574
Specificity						
rE2-CHIKV ELISA	0.9665		0.90	26.541, 1.885	0.7904	0.6841–0.8793
EIA-ICC	0.9850		0.90	18.876, 1.272	0.5559	0.4340 - 0.6740
Commercial Assay	0.9411		0.90	55.179, 4.391	0.9421	0.8829–0.9788

^a^UL (upper limit): value considered (with 95% confidence) to be the highest possible value for that parameter

^b^LL (lower limit): value considered (with 95% confidence) to be the lowest possible value for that parameter

## Discussion

Techniques used for diagnosis of infections by CHIKV in the acute viremic phase include CHIKV isolation in cell culture, detection of viral antigens by enzyme immunoassay, immunofluorescence, and detection of the viral genome by RT-qPCR. However, these methods have reduced usefulness after the first week of disease [16, 18–20]. Alternatively, detection of specific antibodies against CHIKV is used for diagnosis after the acute phase. However, a major problem for serologic diagnosis of alphaviruses (including CHIKV) is the cross-reactivity of antibodies to common antigens of viruses of the genus [21, 22]. In the present study, we have used as ELISA antigen a recombinant E2 protein of CHIKV produced in *E. coli*. The production of recombinant antigen in *E. coli* allows the obtainment of high amounts of the viral product without contamination risk by viral handling. It is known that the E2 protein of alphaviruses is involved in binding and entry of the virus in host cells [23–25]. Besides that, E2 of CHIKV is an important viral antigen that induces neutralizing antibodies for host protection [26–28]. Hence, E2 protein contains suitable antigens for use in diagnostic serologic tests and also could be used as a potential vaccine candidate against CHIKV infection.

Testing human sera by rE2-CHIKV ELISA, we observed high antibody titer levels anti-CHIKV (1600 to 12,800), suggesting it may detect even low levels of anti-CHIKV antibodies present in the samples. On the other side, the rE2-CHIKV ELISA did not show any cross-reactivity signal with the polyclonal mouse hyperimmune serum anti-MAYV that is also a virus genetically grouped in the same *Alphavirus* Semliki Forest group of CHIKV [29]. The differential diagnosis of CHIKV cases with MAYV is important in Brazil and neighbor countries because both can produce a disease with similar symptoms [30]. Furthermore, cross-reactions to hyperimmune sera against other alphaviruses were not observed using the rE2-CHIKV ELISA, suggesting it is a CHIKV specific test that could overcome cross-reactions. These findings encourage the usage of rE2-CHIKV ELISA as a routine test for analysis of a high number of sera from patients with acute febrile illnesses discriminating those infected by CHIKV from infections by other arboviruses.

After the reemergence and spreading of CHIKV around the world, several assays have been developed to detect antigens and antibodies for CHIKV infection diagnosis [31]. Many of these serologic methods use viral antigens from cell culture and the virus in neutralization tests [32–35]. Fifty-nine human serum samples from CHIKV suspected cases were used to evaluate the performance of both IgG and IgM rE2-CHIKV ELISAs. The assay was able to detect 44% (26/59) IgG positive samples. These results, when compared to those obtained by PRNT_50_ for CHIKV, a high specific assay, evidenced 89.66% sensitivity and 100% specificity for the IgG rE2-CHIKV ELISA. Moreover, the IgG rE2-CHIKV ELISA presented similar results to those obtained with two other enzyme immunoassays, the EIA-ICC and the commercial CHIKV immunochromatography assay (Lumiquick, USA), with 82.76 and 89.66% sensitivity and 96.67 and 100% specificity, respectively. A proportion of 73% of the IgG positive samples detected by rE2-CHIKV ELISA had high avidity to the rE2 protein, indicating a great affinity of these human serum antibodies to the antigen and corroborating the good efficiency of the assay. We highlight that IgG rE2-CHIKV ELISA described in the present study show several advantages compared to the other serologic assays that use native or recombinant viral antigens, like the EIA-ICC [36], total viral antigens [33], neutralization assay [35] and baculo-expressed proteins [14], which are more laborious and need biosafety specific care due to the handle of live viruses. Therefore, the IgG rE2-CHIKV ELISA could be recommended as a routine test for diagnosis of patients, particularly those with chronic arthropathy as well as for serologic surveys.

Evaluating the fifty-nine human sera by the IgM rE2-CHIKV ELISA, the assay detected 40.6% (24/59) IgM positive samples, demonstrating a high sensitivity (92.48%), superior to that of the EIA-ICC (74.01%) and slightly higher than that of the commercial assay (91.31%). Also, the rE2-CHIKV ELISA presented a high specificity (79.04%), especially when compared to that of the EIA-ICC (55%). However, only 25% of IgM positive sera to CHIKV showed high avidity to the rE2 protein, which could be related to the preferential binding of IgM on epitopes surface of E1-E2 glycoproteins, rather than to the individual E2 [37] or to the relative immaturity of the first produced antibodies in the acute phase of the infection by CHIKV [38]. The IgM detection results of our assay are in agreement with other studies that used rE2-CHIKV for IgM detection [15, 39]. Our results encourage the use of IgM rE2-CHIKV ELISA as a routine test for diagnosis of recent CHIKV infection.

## Conclusions

We have developed a specific and sensitive IgG and IgM assay for CHIKV diagnosis (rE2-CHIKV ELISA). The assay is also rapid, simple, cost-effective and safe. Our results reveal the great potential of this assay for diagnosis of CHIKV infections, which can be used as a routine diagnosis assay of acute or convalescent suspect patients, as well for serologic surveys.

## Methods

### Recombinant antigen

The recombinant antigen sequence of rE2 of CHIKV (strain DRDE-06), comprising 1167 nucleotides (nt) in length, without the transmembrane region (Genbank accession number MG945127), was cloned into a pET-30a plasmid vector and expressed in *E. coli* system with a six-histidine tag at N-terminal portion by Biomatik Corporation (USA). The rE2 was purified under native conditions and its molecular weight, integrity, and purity were assessed by SDS-PAGE and Western blot using mouse monoclonal anti-his antibody conjugated to horseradish peroxidase (Sigma, USA).

### CHIKV ELISA with recombinant antigen E2 protein (rE2-CHIKV ELISA)

The rE2 of CHIKV was used as an antigen in an indirect ELISA for diagnosis of CHIKV infection. Concentrations of rE2, from 0.5 μg/ml to 8 μg/ml, diluted in 0.05 M Carbonate-Bicarbonate buffer pH 9.6 (Sigma-Aldrich, USA) were added to wells of microplates (Corning, USA) and as negative control, *Escherichia coli* cells extract was diluted in same dilution and added to the other half of the plate. Plates were incubated for 18 and 36 h in a wet chamber at 4 °C and washed between each assay step with 150 μl of PBS-T (Phosphate-buffered saline (PBS) with 0.05% (*v*/v) Tween®). After washing three times, plates were blocked for 2 h at 37 °C with 150 μl of 10% (*w*/*v*) non-fat dry milk in PBS-T (blocking solution) to reduce background. In sequence, plates were washed three times and for positive CHIKV IgG, 50 μl of different dilutions (1:100 to 1:800) of a mouse hyperimmune anti CHIKV serum diluted in blocking solution was added to the positive and negative wells. Plates were incubated 1 h at 37 °C and washed four times. After, it was added 50 μl of a 1:2000 horseradish peroxidase (HRP)-conjugated to a goat immunoglobulin anti-mouse IgG (‘Fab’ specific, Sigma-Aldrich, USA) diluted in blocking solution. Plates were incubated 1 h at 37 °C, washed five times and it was added 100 μl of 2,2′-Azinobis [3-ethylbenzothiazoline-6-sulfonic acid]-diammonium salt (ABTS) peroxidase substrate (KPL, USA) per well and incubated for ~ 15 min at 37 °C for test revealing. The plates were read in Titertek Multiscan MMC/340 Microplate Reader at an optical density (O.D.) of 405 nm. ELISA cutoff values were calculated as the mean O.D. of negative controls plus three standard deviations (SDs). All samples were tested in duplicate. Sample with an average O.D. above the cutoff value was considered as positive [40].

### Cross-reactivity in the rE2-CHIKV ELISA

In order to evaluate cross-reactivity of antibodies to alphaviruses in the rE2-CHIKV ELISA, mouse hyperimmune sera to AURV strain BeAr10315, EEEV strain BEAN-1999, MAYV strain BeAr20290, MUCV strain BeAn-8 and WEEV strain SpAn14723 were tested at different dilutions (1:100 to 1:800). These hyperimmune sera had their reactivity previously confirmed by indirect immunofluorescence tests [41] against their reciprocal viruses infecting tissue culture cells, labelling for nuclei, cytoplasm and the virus (Additional file 3: Figure S1).

### Testing human samples with rE2-CHIKV ELISA

A total of 59 serum samples from patients clinically suspected of CHIKV infection were tested by rE2-CHIKV ELISA. Human assays used the HRP-conjugated goat anti-human IgG ‘Fab’ specific (Sigma-Aldrich, USA) diluted at 1:2000 or the HRP-conjugated goat anti-human IgM ‘Fc5μ’ (Merck Millipore, USA) diluted at 1:25000. Positive samples were diluted from 1:100 to 1:25,600.

### Other serological tests used for diagnosis of infection by CHIKV

Results obtained for the 59 human sera in IgG and IgM rE2-CHIKV ELISAs were compared to those obtained by an in-house EIA-ICC using C6/36 cells [42] and also to those obtained by a commercial immunochromatographic test for quick detection of anti-CHIKV IgG and IgM antibodies (Lumiquick, USA).

### Plaque reduction neutralization test

To evaluate the specificity of anti-CHIKV antibodies in human serum samples, a PRNT_50_ was performed in Vero cells. Briefly, human sera previously inactivated were serially diluted (1/10 to 1/20,480) in Dulbecco’s Modified Eagle’s Medium (DMEM) (Vitrocell, Brazil). Subsequently, 1.75 × 10^2^ PFU of CHIKV, strain BzH1, cordially provided by professor Benedito Antônio Lopes da Fonseca, were mixed with each serum and these mixtures were incubated for 1 h at 37 °C. Then, 200 μl of each mixture was inoculated into Vero cell monolayers in 6 well plates. Cells were incubated 1 h at 37 °C under gently rocking. In sequence, 2 ml of pre-warmed DMEM containing 1% agar (Sigma, USA) and 3% fetal bovine serum (FBS) (Vitrocell, Brazil) were added to each cell monolayer well, and plates were incubated for 2 days at 37 °C at 5% CO_2_ atmosphere. Finally, the cells were fixed in the wells with 4% formaldehyde (Labsynth, Brazil) solution for 2 h and stained with 1% crystal violet (Merck, USA) during 5 min, for plaque visualization. Plaque reduction was calculated for each serum by comparing their respective number of plaques to the positive control, which was inoculated with 1.75 × 10^2^ PFU of CHIKV. The cut off titer for positivity was set as 80.

### rE2-CHIKV ELISA antibody avidity assay

Human IgG and IgM positive samples in the rE2-CHIKV ELISA were submitted to an avidity assay [43]. Briefly, after primary antibody incubation, 100 μl of a 6 M urea solution diluted in PBS or only PBS was added to the wells, and the plates were incubated for 10 min at 37 °C in a wet chamber. Then, plates were washed four times with PBS-T. The subsequent steps of the rE2-CHIKV ELISA were performed as described above. Relative avidity index (RAI) was calculated for each sample by dividing the liquid O.D. in urea treated wells by those in untreated wells (PBS), and proportions were showed in percentage. Samples with RAI > 60% were considered as having high avidity, 40 to 60% as medium avidity, and < 40% as low avidity.

### Bayesian analysis

The sensitivity and specificity of rE2-CHIKV ELISA, EIA-ICC and immunochromatographic test for IgM detection were estimated by the Bayesian method introduced by Joseph et al. [44]. The Bayesian method allows us to estimate the accuracy of the test in the absence of a gold standard, by incorporating into the analysis prior knowledge about the performance measures. Prior information about test sensitivities and specificities was obtained from the literature [13, 15, 36] and data available from LumiQuick Inc. (Santa Clara, USA), and it was represented by the use of beta (a,b) probability distributions, where the values of “a” and “b” determine the shape of the curve. These values were assessed using the function epi.betabuster of the epiR package in R software. This function calculates the values “a” and “b” based on a prior estimate and a 100(p)% uncertainty range, defined by a lower (LL) or upper limit (UL). Bayesian analyses were based on Markov chain Monte Carlo (MCMC) computations and performed with OpenBUGS (Imperial College and MRC, United Kingdom, available at www.mrc-bsu.cam.ac.uk/software/bugs). Posterior inferences were based on summaries of 1,000,000 iterations with a sampling lag of 10, after a burn-in of 10,000 iterations. The final results were presented as a median and a 95% probability interval (PI, percentiles 2.5 and 97.5) of each parameter estimate.

## Additional files

Additional file 1: **Table S1.** Detection results of human samples obtained by rE2-CHIKV ELISA, EIA-ICC, CHIKV commercial assay and PRNT_50._ Bold or POS.: positive sample, ND: Not detected. (DOCX 18 kb)

Additional file 2: **Table S2.** The relative avidity index for IgG and IgM of each sample. Samples with RAI > = 60% were considered of High Avidity, between 40 and 60% of Medium Avidity and = < 40% of Low Avidity. ND: Not detected. (DOCX 15 kb)

Additional file 3: **Figure S1.** Immunofluorescence assay of infected cells. Specific homotypic antibodies detection by infected Vero cells to the respectively hyperimmune sera of alphaviruses. Green: hyperimmune sera labeling viruses; Blue: Nuclei, Red: Cytoplasm; Pos.: Positive infected cells and Neg.: Negative non-infected cells. (PDF 599 kb)

## Abbreviations

AURV: Aura virus
CHIKV: Chikungunya virus
DMEM: Dulbecco’s Modified Eagle’s Medium
EEEV: Eastern equine encephalitis virus
EIA-ICC: Enzyme immunoassay using infected cultured cells
ELISA: Enzyme-linked immunosorbend assay
FBS: Fetal Bovine Serum
HRP: Horsedish peroxidase
IgG: Immunoglobulin G
IgM: Immunoglobulin M
kDa: kilodalton
Mac-ELISA: IgM capture enzyme-linked immunosorbend assay
MAYV: Mayaro virus
MUCV: Mucambo virus
O.D.: Optical Density
ORF: Open reading frame
PBS: Phosphate-buffered saline
PBS-T: Phosphate-buffered saline with Tween
PFU: Plaque forming unit
PRNT_50_: Plaque reduction neutralization test of 50% of plaques
RAI: Relative avidity index
rE2: recombinant envelope protein 2
RNA: Ribonucleic acid
RT-qPCR: Real time quantitative polymerase chain reaction
SDS-Page: Sodium dodecyl sulfate polyacrylamide gel electrophoresis
WEEV: Western equine encephalitis virus
