# Supplementary material for: Enzyme-linked immunosorbent assay using recombinant envelope protein 2 antigen for diagnosis of Chikungunya virus
Source: Virol J. 2018 Jul 24;15:112. doi: 10.1186/s12985-018-1028-1 (PMC6056935; doi:10.1186/s12985-018-1028-1)
Supplement: Supplementary file 1 — Table S1. Detection results of human samples obtained by rE2-CHIKV ELISA, EIA-ICC, CHIKV commercial assay and PRNT50. Bold or POS.: positive sample, ND: Not detected. (DOCX 18 kb) [file 12985_2018_1028_MOESM1_ESM.docx]

| Sample number | IgG detection | | | PRNT_50_ | IgM detection | | |
| --- | --- | --- | --- | --- | --- | --- | --- |
|  | rE2-ELISA | EIA-ICC | Commercial Assay |  | rE2-ELISA | EIA-ICC | Commercial Assay |
| 1 | ND | ND | ND | <10 | **POS.** | **POS.** | ND |
| 2 | **3200** | **100** | **POS.** | **1280** | **POS.** | **POS.** | ND |
| 3 | **3200** | **100** | **POS.** | **2560** | **POS.** | **POS.** | ND |
| 4 | ND | ND | ND | <10 | **POS.** | ND | ND |
| 5 | **6400** | **200** | **POS.** | **5120** | **POS.** | **POS.** | **POS.** |
| 6 | **6400** | **200** | **POS.** | **1280** | **POS.** | **POS.** | ND |
| 7 | **6400** | **200** | **POS.** | **5120** | ND | **POS.** | ND |
| 8 | **1600** | **200** | **POS.** | **1280** | **POS.** | **POS.** | ND |
| 9 | **1600** | **200** | **POS.** | **2560** | **POS.** | **POS.** | ND |
| 10 | ND | ND | ND | <10 | ND | **POS.** | ND |
| 11 | ND | ND | ND | <10 | **POS.** | **POS.** | ND |
| 12 | **400** | ND | **POS.** | **1280** | **POS.** | **POS.** | **POS.** |
| 13 | **800** | **200** | **POS.** | **2560** | **POS.** | **POS.** | ND |
| 14 | **3200** | **400** | **POS.** | **5120** | **POS.** | ND | ND |
| 15 | **1600** | **200** | **POS.** | **2560** | **POS.** | **POS.** | ND |
| 16 | **100** | **100** | **POS.** | **2560** | **POS.** | **POS.** | **POS.** |
| 17 | **3200** | **200** | **POS.** | **1280** | **POS.** | **POS.** | **POS.** |
| 18 | **1600** | **100** | ND | 640 | ND | ND | ND |
| 19 | ND | ND | ND | <10 | ND | **POS.** | ND |
| 20 | **12800** | **400** | **POS.** | **10240** | ND | **POS.** | ND |
| 21 | ND | ND | ND | <10 | **POS.** | **POS.** | ND |
| 22 | **1600** | **200** | **POS.** | **1280** | **POS.** | **POS.** | ND |
| 23 | **3200** | **800** | **POS.** | **20480** | ND | **POS.** | ND |
| 24 | **1600** | **800** | **POS.** | **5120** | **POS.** | **POS.** | ND |
| 25 | ND | ND | ND | <10 | **POS.** | ND | ND |
| 26 | **400** | **100** | **POS.** | **1280** | **POS.** | ND | ND |
| 27 | ND | ND | **POS.** | **5120** | ND | ND | **POS.** |
| 28 | **400** | **100** | **POS.** | **2560** | **POS.** | ND | **POS.** |
| 29 | **1600** | **400** | **POS.** | **2560** | **POS.** | ND | ND |
| 30 | **1600** | ND | **POS.** | **5120** | **POS.** | **POS.** | **POS.** |
| 31 | **1600** | **200** | **POS.** | **5120** | **POS.** | **POS.** | **POS.** |
| 32 | **400** | **100** | **POS.** | **2560** | **POS.** | **POS.** | **POS.** |
| 33 | ND | ND | ND | <10 | **POS.** | ND | ND |
| 34 | ND | ND | ND | **80** | **POS.** | ND | ND |
| 35 | ND | ND | ND | **80** | **POS.** | **POS.** | **POS.** |
| 36 | **200** | **100** | **POS.** | **5120** | **POS.** | ND | **POS.** |
| 37 | **1600** | **400** | **POS.** | **2560** | **POS.** | ND | ND |
| 38 | ND | ND | ND | <10 | ND | **POS.** | ND |
| 39 | ND | **100** | ND | <10 | ND | **POS.** | ND |
| 40 | ND | ND | ND | <10 | ND | **POS.** | ND |
| 41 | ND | ND | ND | <10 | ND | **POS.** | ND |
| 42 | ND | ND | ND | <10 | ND | **POS.** | ND |
| 43 | ND | ND | ND | <10 | ND | **POS.** | ND |
| 44 | ND | ND | ND | <10 | **POS.** | **POS.** | ND |
| 45 | ND | ND | ND | <10 | ND | **POS.** | ND |
| 46 | ND | ND | ND | <10 | **POS.** | **POS.** | ND |
| 47 | ND | ND | ND | <10 | ND | **POS.** | ND |
| 48 | ND | ND | ND | <10 | ND | **POS.** | ND |
| 49 | ND | ND | ND | <10 | ND | **POS.** | ND |
| 50 | ND | ND | ND | <10 | ND | ND | ND |
| 51 | ND | ND | ND | <10 | ND | ND | ND |
| 52 | ND | ND | ND | <10 | ND | ND | ND |
| 53 | ND | ND | ND | <10 | **POS.** | ND | ND |
| 54 | ND | ND | ND | <10 | ND | ND | ND |
| 55 | ND | ND | ND | <10 | ND | ND | ND |
| 56 | ND | ND | ND | <10 | **POS.** | ND | ND |
| 57 | ND | ND | ND | <10 | ND | ND | ND |
| 58 | ND | ND | ND | <10 | ND | ND | ND |
| 59 | ND | ND | ND | <10 | ND | ND | ND |
