# Supplementary material for: Enzyme-linked immunosorbent assay using recombinant envelope protein 2 antigen for diagnosis of Chikungunya virus
Source: Virol J. 2018 Jul 24;15:112. doi: 10.1186/s12985-018-1028-1 (PMC6056935; doi:10.1186/s12985-018-1028-1)
Supplement: Supplementary file 2 — Table S2. The relative avidity index for IgG and IgM of each sample. Samples with RAI > = 60% were considered of High Avidity, between 40 and 60% of Medium Avidity and = < 40% of Low Avidity. ND: Not detected. (DOCX 15 kb) [file 12985_2018_1028_MOESM2_ESM.docx]

| Serum sample number | Relative IgG avidity index (RAI) | IgG avidity Classification | Relative IgM avidity index (RAI) | IgM avidity Classification |
| --- | --- | --- | --- | --- |
| 1 | - | ND | 58.13% | Medium Avidity |
| 2 | 70.23% | High Avidity | 65.38% | High Avidity |
| 3 | 89.09% | High Avidity | - | ND |
| 5 | 95.29% | High Avidity | 31.36% | Low Avidity |
| 6 | 77.93% | High Avidity | - | ND |
| 7 | 92.85% | High Avidity | - | ND |
| 8 | 100.31% | High Avidity | - | ND |
| 9 | 65.57% | High Avidity | 40.47% | Medium Avidity |
| 12 | 83.33% | High Avidity | 40.47% | Medium Avidity |
| 13 | 27.27% | Low Avidity | 17.81% | Low Avidity |
| 14 | 93.36% | High Avidity | 20.18% | Low Avidity |
| 15 | 63.84% | High Avidity | 50.34% | Medium Avidity |
| 16 | 33.08% | Low Avidity | 49.56% | Medium Avidity |
| 17 | 77.01% | High Avidity | - | ND |
| 18 | 69.78% | High Avidity | - | ND |
| 20 | 107.48% | High Avidity | - | ND |
| 22 | 77.04% | High Avidity | 92.71% | High Avidity |
| 23 | 86.39% | High Avidity | - | ND |
| 24 | 77.63% | High Avidity | - | ND |
| 25 | - | ND | 48.65% | Medium Avidity |
| 26 | 77.78% | High Avidity | 26.12% | Low Avidity |
| 28 | 14.41% | Low Avidity | 15.00% | Low Avidity |
| 29 | 94.72% | High Avidity | 24.65% | Low Avidity |
| 30 | 53.28% | Medium Avidity | 38.64% | Low Avidity |
| 31 | 32.84% | Low Avidity | 24.23% | Low Avidity |
| 32 | 38.89% | Low Avidity | 54.79% | Medium Avidity |
| 33 | - | ND | 23.63% | Low Avidity |
| 34 | - | ND | 61.47% | High Avidity |
| 35 | - | ND | 24.95% | Low Avidity |
| 36 | 54.30% | Medium Avidity | 24.69% | Low Avidity |
| 37 | 98.45% | High Avidity | - | ND |
| 44 | - | ND | 118.61% | High Avidity |
| 53 | - | ND | 60.26% | High Avidity |
| 56 | - | ND | 73.68% | High Avidity |
