# Supplementary figures and images for: Enzyme-linked immunosorbent assay using recombinant envelope protein 2 antigen for diagnosis of Chikungunya virus
Source: Virol J. 2018 Jul 24;15:112. doi: 10.1186/s12985-018-1028-1 (PMC6056935; doi:10.1186/s12985-018-1028-1)

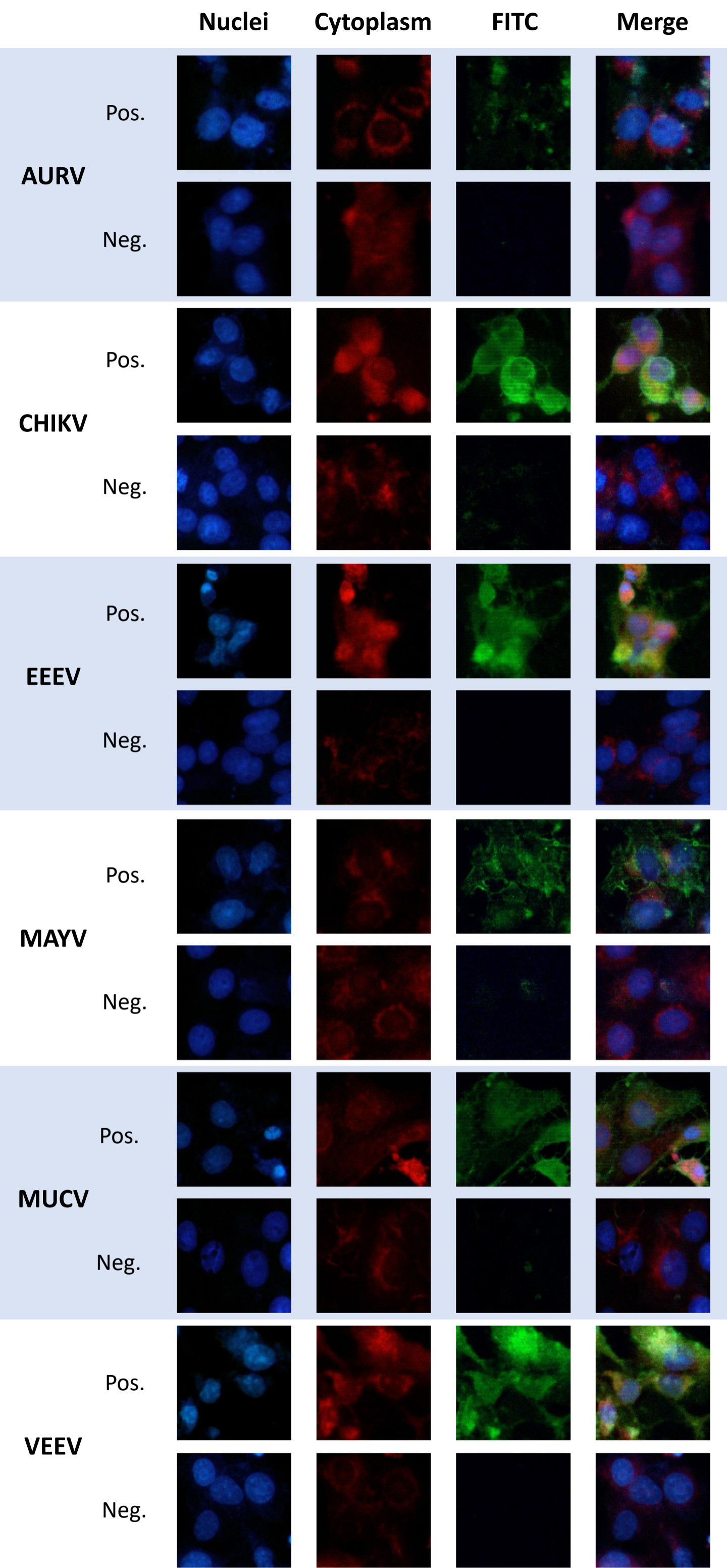

20 μm

Supplement: Supplementary file 3 — Figure S1. Immunofluorescence assay of infected cells. Specific homotypic antibodies detection by infected Vero cells to the respectively hyperimmune sera of alphaviruses. Green: hyperimmune sera labeling viruses; Blue: Nuclei, Red: Cytoplasm; Pos.: Positive infected cells and Neg.: Negative non-infected cells. (PDF 599 kb) [file 12985_2018_1028_MOESM3_ESM.pdf]
